# END-OF-LIFE CARE PREFERENCES AND WELL-BEING IN LONG-TERM CARE RESIDENTS

**DOI:** 10.1093/geroni/igz038.2474

**Published:** 2019-11-08

**Authors:** Kelly Shryock, Jacinta Dickens, Anisha Thomas, Suzanne Meeks

**Affiliations:** 1 University of Louisville, Louisville, Kentucky, United States; 2 John Hopkins University, Baltimore, Maryland, United States

## Abstract

Research on end-of-life care in nursing homes comes largely from the viewpoint of staff or family members. We examined patient perspectives on end-of-life care, preferences for care, and quality of life in long-term care settings. We hypothesized that fulfillment of the Self Determination Theory (SDT) needs of autonomy, competence, and relatedness would be related to better well-being and that the degree to which end-of-life care preferences are seen as possible in the setting would be related to SDT need fulfillment and well-being. Preliminary data, collected from older individuals at the end of life (over 55, presence of significant chronic disease, in long term care setting) (n= 72), demonstrated that autonomy, competence, and relatedness measures were moderately and significantly correlated with well-being as measured by life satisfaction, higher positive affect, lower negative affect, and overall quality of life measures The degree to which residents believed that their end-of-life care preferences could be honored in the setting was also significantly correlated with autonomy, competence, relatedness, positive affect, and psychological quality of life. These results are consistent with SDT and suggest that if long term care settings can promote autonomy, connection, and competence in making end of life decisions, possibly by discovering and fulfilling preferences for end of life care, individuals who end their lives on those settings have potential for greater satisfaction and happiness. These results suggest that SDT is a useful framework for ongoing research on how to improve the end of life experiences of older adults in long term care.

